# Envisioning a Hopeful Future Despite Schizophrenia: The Role of Self‐Concept Clarity and Narrative Identity

**DOI:** 10.1111/sjop.70113

**Published:** 2026-05-08

**Authors:** Majse Lind, Ragnhild Svendsen, Kristina Flesjø, Kristine Kahr Nilsson

**Affiliations:** ^1^ Department of Culture & Communication Aalborg University Aalborg Denmark

**Keywords:** hope, narrative identity, recovery, schizophrenia, self‐concept clarity

## Abstract

Schizophrenia (SZ) has been linked with a pessimistic future outlook but the specific factors influencing such outlook are still to be mapped out. SZ is often referred to as a self‐disorder and sense of self has been suggested to be a crucial factor influencing future view. Using a mixed‐measurement approach, the present study examined two aspects of temporal sense of self in SZ—self‐concept clarity and narrative identity—and tested whether each uniquely contributed to predicting future outlook (i.e., levels of future hope and the perceived influence of diagnosis on one's future). A total of 130 adults with SZ participated in the study (*M*_age_ = 34.58, SD = 10.22). Self‐concept clarity was assessed through self‐report and narrative identity through open‐ended elaborations on the future that were content coded following gold‐standard procedures. Hope for the future was assessed using self‐report and perceived influence of diagnosis in the future was assessed through content coded, open‐ended elaborations. Both self‐concept clarity and narrative identity contributed independently to a more hopeful future outlook while self‐concept clarity alone impacted whether diagnosis was expected to influence the future. Distinct dimensions of the temporal sense of self were associated with future outlook in schizophrenia, and their potential role in the recovery process was discussed.

## Keypoints

- In Schizophrenia, both self‐concept clarity and narrative identity independently predicted a more hopeful future outlook, highlighting the importance of a multidimensional temporal sense of self.
- While both aspects of sense of self supported hope, self‐concept clarity uniquely influenced whether individuals expected their diagnosis to shape their future, suggesting distinct psychological mechanisms.
- Findings indicate that targeting both how individuals understand themselves (clarity) and how they construct their life stories (narrative identity) may be important pathways for improving future outlook and supporting recovery in schizophrenia.

Schizophrenia (SZ) is one of the most disabling mental illnesses characterized by a combination of positive symptoms (e.g., hallucinations, delusions) and negative symptoms (e.g., anhedonia, avolition) (Andreasen and Flaum 1991), resulting in high societal costs, primarily through healthcare and productivity costs (Jin and Mosweu 2017). SZ also place a severe burden on families (Magliano et al. 2005), and cause serious personal suffering reflected in low subjective well‐being and declined physical health (Dong et al. 2019) as well as functional impairment (e.g., McCutcheon et al. 2020). As clinical recovery of symptoms and functioning has sadly plateaued in recent years despite major changes and initiatives in treatment options (see Jääskeläinen et al. 2013) personal recovery has gained increasingly attention. Personal recovery is the process in which individuals develop new ways of understanding themselves, maintain hope, and work toward meaningful goals and a fulfilling life despite ongoing symptoms or disability (Leamy et al. 2011; Rossi et al. 2022). Thus, hope is considered a critical components in the rehabilitation and personal recovery of individuals with SZ (Barut et al. 2016; Hayes et al. 2017; Martinez et al. 2023). Various studies have examined the importance of hope for the outcomes of SZ. It has, for instance, been found that individuals with SZ who hold more defeatist beliefs about their future (e.g., the belief that one will eventually fail at goal‐directed behaviors) tend to have more functional impairment and more negative symptoms (Campellone et al. 2016; Grant and Beck 2009). Hopelessness about the future has also been associated with more depressive symptoms, greater risk of suicidal behavior (Berardelli et al. 2019), and hindered clinical recovery in SZ (Martinez et al. 2023). Conversely, hope is recognized as an important factor in personal recovery and quality of life (Choe 2014). Thus, subjective interpretations about the future emerge as a topic of considerable importance. While several studies have examined hope as a predictor of outcomes in SZ, there is limited research investigating hope as the outcome in its own right. Thus, it is still unclear which factors and processes shape how individuals with SZ perceive their future. In order to facilitate recovery processes, which often involve individuals having at least some degree of hope (Barut et al. 2016; Hayes et al. 2017), it is important to understand which factors influence hope. So far, research does not suggest that any typical demographic factors are predictive of hope among patients with SZ (Oles et al. 2015). However, an understudied area that appears highly relevant to hope in SZ, and personal recovery more broadly, is the sense of self. There are both theoretical and empirical grounds for this. Theoretically, disordered selfhood has been proposed as the core dysfunction in SZ that manifest across different levels of the self (e.g., Postmes et al. 2014). At the most basic level, the influential Ipseity‐Disturbance Model (IDM) suggests that SZ involves a disruption of the fundamental sense of ‘me‐ness’ in experience (Lysaker and Lysaker 2010; Sass and Parnas 2003). This disruption manifest in anomalous attention and meaning‐making of one's own thoughts and sensations (hyperreflexivity), a weakened sense of being the subject of one's thoughts and actions (diminished self‐presence), and a detachment from the physical world or reality (disconnect from reality), which also contribute to the hallucinations and delusions. This fundamental disturbance in self may also permeate to higher‐order levels of self, often described as the narrative or social self (Sass and Parnas 2003) which encompasses identity, self‐esteem, self‐image, and personal history (Nelson et al. 2014). For instance, experiencing that ones throught are fragmented and perhaps controlled by outside forces (as is often the case with some halluciations and delutions), makes it difficult to maintain a coherent sense of self through time. Supporting this speculation, research indicates that individuals with SZ generally experience diminished self‐concept clarity (Cicero 2018).

Self‐concept clarity refers to the degree to which individual's self‐concept, including beliefs, attitudes, and values, is clearly defined, internally coherent, and stable over time (Campbell et al. 1996). As such, self‐concept clarity captures a temporal aspect of the self that relates to how individuals perceive themselves across time. A coherent sense of past and present self has been proposed as crucial for adaptive, future‐oriented behavior (Lind et al. 2019; Stinson et al. 2008). Without the perception of a coherent self, it may be difficult to envision and be hopeful about one's future. Converging with this reasoning, a recent study found that lower self‐concept clarity was associated with reduced hope for the future in patients with schizophrenia (Svendsen et al. 2025).

As outlined above, there are both theoretical and empirical grounds for assuming that temporal aspects of the self—how one perceive oneself through time—plays an important role for experiencing a sense of hope in SZ. Note that the “*temporal self*” refers to self‐experience in the context of time (i.e., integration of past, present, and future), and does not imply that temporal processes are empirically examined over time. Besides self‐concept clarity, narrative identity is another construct capturing temporality of the self that may also be important to understanding hope in SZ.

Narrative identity refers to the inner and dynamic story a person crafts about the personal, past, present, and presumed future (McAdams 2018). By organizing life events into a meaningful narrative, it scaffolds self‐continuity and provides a sense of “who I was, who I am, and who I might become” (McAdams 2001). Narratives characterized by a positive tone and themes of agency (autonomy, mastery) and communion (love, belongingness) are consistently associated with higher well‐being (Adler et al. 2016), whereas psychopathology is often linked with disturbed narrative identities (Lind et al. 2020). In SZ, narratives are often marked by negative interpretations of the past, diminished agency (e.g., victimization, lack of control), and thwarted communion (e.g., loneliness, isolation), compared to non‐clinical controls (Cowan et al. 2021; Holm et al. 2016; 2018; Jensen et al. 2021). While the majority of such studies have focused on past narratives, much less work has focused on future component of narrative identity. The few studies that do exist indicate that individuals at high risk for SZ generate shorter and less goal‐directed future narratives (Hazan et al. 2019) and that patients with SZ produce fewer and less extended future chapters than controls (Jensen et al. 2020). Having such anomoulous future narratives are likely also to affects whether they have a positive and hopeful outlook on the future. This has, however, not been tested empirically. Thus, it is still unclear whether narrative identity potentially is related to hope or lack hereof in SZ. It is similarly unclear whether it contributes to hope over and above other aspects of the self, such as self‐concept clarity which has been found to predict hope in SZ (Svendsen et al. 2025). Both of these constructs relate to essentially different aspects of how the self is conceived through time. As described within McAdams' influential three‐level model, self‐concept clarity is situated within the second level of characteristic adaptations (encompassing beliefs, goals, and coping strategies), whereas narrative identity is located at the third level, the life story (McAdams 2018). Methodologically, the two are also approached differently: self‐concept clarity is typically measured via self‐report, while narrative identity is assessed through content coding of phenomenologically rich narratives (Adler et al. 2017). It is therefore likely that they tap into different aspects of the self that potentially may shape future outlook and hope among individuals with SZ.

## 1 The Present Study

Taken together, prior research suggests that individuals with SZ report unclear self‐concepts and construct past narrative identities marked by negative valence, diminished agency (e.g., victimization, lack of control), and thwarted communion (e.g., loneliness, isolation). In contrast, future aspects of narrative identity remain understudied, and their links to broader future outlook are therefore unclear. While self‐concept clarity has been linked with hope, the same does not apply to narrative identity. Thus, to contribute toward filling this void in the empirical literature, the present study examined two theoretically distinct aspects of the temporal self—narrative identity and self‐concept clarity—to explore their unique contributions to future outlook and hope in SZ. To our knowledge these constructs have not been examined in tandem in relation to hope and future outlook.

Since these constructs capture different levels of the temporal self (McAdams 2018), we expected them to relate uniquely to how individuals with SZ perceive their future. When assessing future outlook, we focus on two central dimensions: (1) hope (Choe 2014) and (2) the perceived influence of diagnosis on the future. For many individuals with severe and chronic mental illness, the diagnosis itself becomes a key reference point when imagining what lies ahead. Prior research shows that such diagnoses profoundly shape identity, influencing roles, attitudes, and expectations for the future (Yanos et al. 2010). Accordingly, we define a positive future outlook as one characterized not only by hope but also by a diminished dominance of the diagnosis in shaping future possibilities.

Based on findings by Svendsen et al. (2025), we expected self‐concept clarity to be associated with hope in future outlook. As self‐efficacy, perceived control, and social support have been linked to hope in individuals with mental illness (Hodges et al. 2004), and conceptually overlap with narrative themes of agency (e.g., mastery, autonomy) and communion (e.g., connectedness, belonging), we expected these themes to predict hope as well. The hypotheses were the following: (1) Future narrative identity characterized by a positive tone and themes of agency and communion is associated with a more hopeful outlook in which the diagnosis is less influential. (2) Higher self‐concept clarity is associated with a more hopeful and less illness‐centered future outlook. (3) Given their theoretically distinctness, both constructs will show predictive value above and beyond each other and the correlation between them will be significant but small. Finally, we expect these associations to remain after controlling for demographics (age and gender) as well as illness severity (age of onset and overall functioning).

## 2 Methods

### 2.1 Participants

The present investigation is part of a study conducted by Svendsen et al. (2025). This study included a total of 130 adults diagnosed with SZ (*M* = 34.58, SD = 10.22, women = 85.4%). Within this sample, 51 (39.2%) had completed a tertiary education. On average, participants were 23.72 years (SD = 7.02) when receiving the diagnosis and have, on average, lived with the diagnosis for more than a decade (*M*_years_ = 10.96 years, SD = 8.02). A total of 20.8% were hospitalized within the past 6 months, 3.8% within the last 6–12 months, 10% within the last 12–24 months, 46.2% within the past 2 years, 3.1% were currently hospitalized and another 16.2% had never been hospitalized (see Table 1).

**TABLE 1:** Descriptive statistics of the sample in terms of sociodemographics and illness course variables.

	Mean (SD)	*n* (%)
Age—years	34.58 (10.22)	
Gender (female)		111/85.4%
Male		18 (13.8%)
Other		1 (0.8%)
Completed education		51 (39.2%)
No education		79 (60.8%)
Age at diagnosis	23.72 (7.02)	
Illness duration in years	10.96 (8.02)	
Self‐concept clarity (SCCS)	40.03 (9.63)	
Functional impairments (WSAS)	22.10 (9.44)	
Hope (SHS)	11.06 (5.85)	

Abbreviations: SCCS, Self‐Concept Clarity Scale (SCCS; Campbell et al. 1996); SHS‐9, Schizophrenia Hope Scale (Choe 2014); WSAS, Work and Social Adjustment Scale (Mundt et al. 2002).

### 2.2 Procedure

Participants were recruited from the nationwide mental‐health organization named “EN AF OS” (2022) under the Danish Health Authorities. This organization specifically targets the stigmatization of mental disorders and promotes the inclusion of individuals with mental disorders. Individuals with mental disorders are affiliated with this organization. The study design was first piloted in a lived experience panel with representatives of the subgroup with SZ who commented upon the study design. After minor adjustments to the design, an electronic link to the study was distributed through the organization. Inclusion criteria were a minimum age of 18 and a diagnosis of SZ as established by qualified doctors with psychiatric training. In Denmark all doctors are required to follow the national guidelines for diagnosing and treating SZ (Sundhedsstyrelsen 2018), with the intention of ensuring uniform standards and procedures and that a diagnosis of SZ diagnosis correspond to the criteria outlined in the International Classification of Diseases—with the 10th version in effect at the time of this study (WHO 1993). Informed consent was obtained from all the participants, and data were treated in accordance with the Danish Data Protection Act. The recruitment of participants was conducted during a confined period of 1 week, where permission to recruit participants through the mental health organization was granted.

### 2.3 Measures

#### 2.3.1 Future Outlook

*The Schizophrenia Hope Scale* (SHS‐9; Choe 2014) was used to measure hope for the future. The SHS‐9 contains nine statements about the future. An example of an item is: “I will be happy in the future”. The respondents are asked to rate how well each statement describes how they see themselves in the future. The SHS‐9 yields a total score between 0 and 18, with higher scores indicating more hope. A Danish version of the SHS‐9 was used. The SHS‐9 has in other studies shown good internal consistency, test–retest reliability as well as solid convergent and divergent validity (Choe 2014; Choe et al. 2020). In this study, the internal consistency of the SHS‐9 was high (Cronbach's *α* = 0.93).

*Perceived Influence of Diagnosis on the Future*. Participants were asked to provide open‐ended answers to the question: “*Does your schizophrenia diagnosis influence how you see your future?*” Thus, the question captures an important dimension of future outlook that is not addressed by the SHS‐9 alone. The elaboration related to whether the diagnosis would influence the future was coded on a 3‐point Likert Scale with 1 = *no influence on the future*, 2 = *some influence on the future*, and 3 = certainly *influence on the future*. Following standardized coding procedures, two blinded raters coded 20% of all future statements. Based on excellent inter‐rater reliability (*κ* = 0.75), the remaining narratives were coded by one of the coders.

### 2.4 Temporal Sense of Self

*Self‐Concept Clarity: The Self‐Concept Clarity Scale* (SCCS; Campbell et al. 1996) is a widely used measure to capture self‐concept clarity. The Danish translation of the scale has demonstrated adequate psychometric properties (Holm and Thomsen 2018). The SSCS contains 12 statements, each rated on a five‐point Likert scale ranging from 1 = *strongly agree* to 5 = *strongly disagree*, “when I think about the kind of person I have been in the past, I'm not sure what I was really like.” The SCCS yields a total score of 12–60 and scores were arranged such that higher scores indicated less self‐concept clarity. In the present study, the internal consistency for SCSS was good (Cronbach's *α* = 0.88).

*Future Narrative Identity*. Participants were asked to elaborate on an open‐ended question about their future: The elaboration was coded for emotional tone, agency, and communion following McAdams's (2001) standardized coding manuals. Emotional tone was coded on a 3‐point Likert scale with 1 = *negative*, 2 = *mixed or neutral*, and 3 = *positive tone*. Some participants exemplified a more negative or pessimistic narrative for the future: “In the future I would really like to go to university, but I do not know if I have the bandwidth to actually go. My grades are Bs and As, but I even need my mom's assistance to complete this survey” and “I want to find something that makes my life meaningful. It is hard for me to take initiative and find motivation to do anything. It is a challenge” while other participants' future narratives were induced with more positivity: “I am part of ‘One of us’ which gives me a lot of hope. I also have fantastic friends and family that also induce a lot of hope for the future” and “to live a long life with my boyfriend that I have been together with for eight years. And to continue keeping my job”. Agency (i.e., themes of achievement/responsibility, power/impact, self‐insight, and status/victory) was coded as 1 = present and 0 = absent. Similarly, communion (i.e., themes of love/friendship, dialogue, caring/help, and unit/togetherness) was coded as 1 = present and 0 = absent. Adhering to standardized coding procedures, two raters coded 20% of all future statements. Based on excellent inter‐rater reliability on emotional tone (*κ* = 0.72), agency (*κ* = 0.73), and communion (*κ* = 0.73), the remaining narratives were coded by one, blinded rater.

*Basic sociodemographic and illness‐related data* were also collected as part of the assessment. Sociodemographic variables included age, gender, and tertiary education, defined as any completed education after secondary school. Illness‐related variables included age at onset of schizophrenia and functional impairment, assessed using *the Work and Social Adjustment Scale* (WSAS; Mundt et al. 2002). The WSAS requests the respondents to assess the degree to which their mental disorder affects their ability to function in the five domains (i.e., work, home management, social leisure, private leisure, and personal relationships) on a 9‐point scale ranging from 0 = *no functional impairment* to 8 = *a high degree of functional impairment*. The total score across the five domains provides an overall estimate of functional impairment. The Danish version of the WSAS has been validated in different studies on psychiatric patients with regards to its internal consistency, unidimensional factor structure, and convergent validity (Hovmand et al. 2024; Nilsson 2012; Nilsson et al. 2012). The WSAS showed good internal consistency (Cronbach's *α* = 0.85). Earlier onset has been linked to more severe symptoms, poorer functioning, and less favorable life outcomes (Immonen et al. 2017; Jaracz et al. 2007) and thus may negatively influence individuals' ability to envision a positive future. In addition, functional impairment has been found to impact self‐perception in SZ (Sarraf et al. 2022), and may therefore also influence perception of future outlook.

### 2.5 Data‐Analytic Strategy

Pearson's *r* correlations were performed to examine associations between temporal sense of self (i.e., self‐concept clarity & narrative identity) and future outlook (i.e., hope for future & influence of diagnosis in future). Two hierarchical multivariate regression analyses were then conducted to examine the main effects of self‐concept clarity and narrative identity on (1) hope for the future and (2) perceived influence of diagnosis as criterion variables. In step one, age and gender were entered to control for sociodemographic‐specific characteristics and enhance the generalizability to other populations with SZ. In step two, two illness related variables (i.e., age at onset & functional impairment), considered competing predictors of the outcomes, were added to the model. Entering these control variables before the primary predictors ensured that any variance explained by the temporal self‐constructs in the final step was above and beyond the effects of demographics and illness‐related factors. Finally, in Step 3, the two aspects of the temporal self (i.e., self‐concept clarity and narrative identity) were added to the model to assess their main effects. For parsimony, only aspects of the temporal self that were significantly related to the criterion variables in the bivariate correlational analyses were entered at this step. All the analyses were computed using SPSS—version 29.020.

## 3 Results

### 3.1 Bivariate Associations Between Main Study Variables

First, higher self‐concept clarity and future narrative identities characterized by a more positive tone and stronger themes of communion were associated with greater hopefulness about the future. These associations were small‐to‐moderate in magnitude, except for tone, which showed a stronger moderate effect. In contrast, themes of narrative agency were not significantly related to hope (see Table 2).

**TABLE 2:** Correlations between main study variables.

	1	2	3	4	5	6
1. Future hope	—	−0.46**	0.07	0.30**	0.57**	−0.34**
2. Influence of diagnosis	—	—	0.14	−0.02	0.00	0.39**
3. Agency	—	—	—	−0.13	0.28**	0.16
4. Communion	—	—	—	—	0.19	0.03
5. Tone	—	—	—	—	—	−0.15
6. Self‐concept clarity	—	—	—	—	—	—

** *p* < 0.01.

Second, higher self‐concept clarity was small‐to‐moderately linked to perceiving the SZ diagnosis as less influential in the future. No aspects of narrative identity were significantly associated with the perceived influence of diagnosis (see Table 2) and were therefore not included in subsequent regression analyses.

Finally, a more hopeful future outlook was itself moderately associated with the diagnosis having less perceived influence in the future. Notably, self‐concept clarity was not significantly associated with narrative identity (tone, communion, agency). Based on these preliminary analyses, self‐concept clarity was included in both hierarchical regressions, whereas emotional tone and communion themes in future narratives were only included in the first regression on hope.

### 3.2 Predictors of Hope for the Future

A hierarchical multiple regression analysis was conducted to examine predictors of future hope. Control variables (age and gender) were entered in Step 1, illness‐related variables (age of onset and general functioning) in Step 2, and temporal sense of self variables (communion, tone, and self‐concept clarity) in Step 3. The model in Step 1 was not significant and explained only a small proportion of variance in future hope (Adj. *R* ^2^ = 0.03). The addition of illness‐related variables in Step 2 significantly improved the model, explaining an additional 19% of the variance, with general functioning emerging as a significant negative predictor (*β* = −0.39, *p* < 0.001). In Step 3, the inclusion of temporal sense of self variables led to a substantial increase in explained variance with the final model explaining 54% of the variance in future hope. Thus, in this final model, tone (*β* = 0.51, *p* < 0.001) and communion (*β* = 0.17, *p* < 0.05) were significant positive predictors, while self‐concept clarity (*β* = −0.22, *p* < 0.05) and general functioning (*β* = −0.22, *p* < 0.01) were significant negative predictors. Age and gender were not significant in the final model (see Table 3).

**TABLE 3:** Hierarchical regressions model predicting hope for future.

Criterion variable	Predictor variables			Step 1 *β*	Step 2 *β*	Step 3 *β*
	Control variables	Illness variables	Temporal sense of self			
Future hope	Age			0.23*	0.12	0.04
	Gender			0.01	0.01	0.12
		Age of onset			0.00	0.06
		General functioning			−0.39***	−0.22**
			Communion			0.17*
			Tone			0.51***
			Self‐concept clarity			−0.22*
Model change (Step 1–Step 3)						
*F*				2.65	5.40***	14.93***
Δ*R* ^2^				0.05	0.14***	0.35***
Adj. *R* ^2^				0.03	0.19	0.54

* *p* < 0.05.

** *p* < 0.01.

*** *p* < 0.001.

### 3.3 Predictors of the Perceived Influence of Diagnosis on the Future

The second hierarchical multiple regression analysis was conducted to examine predictors of the perceived influence of diagnosis on the future. Control variables (age and gender) were entered in Step 1, illness‐related variables (age of onset and general functioning) in Step 2, and self‐concept clarity in Step 3. In Step 1, the model was significant and explained 11% of the variance, with age emerging as a significant negative predictor (*β* = −0.31, *p* < 0.01), indicating that younger participants reported a stronger perceived influence of diagnosis on their future. The addition of illness‐related variables in Step 2 did not significantly improve the model (Adj. *R* ^2^ = 0.13), although general functioning showed a trend‐level association (*β* = 0.19, *p* < 0.06). In Step 3, the inclusion of self‐concept clarity resulted in a small but significant increase in explained variance with the final model explaining 17% of the variance. In this final model, self‐concept clarity was a significant positive predictor (*β* = 0.26, *p* < 0.05), while age showed a trend‐level effect (*β* = −0.27, *p* < 0.06). No other predictors were significant (see Table 4).

**TABLE 4:** Hierarchical regression model predicting the perceived influence of diagnosis on the future.

Criterion variable	Predictor variables			Step 1 *β*	Step 2 *β*	Step 3 *β*
	Control variables	Illness variables	Temporal sense of self			
Perceived influence of diagnosis in the future	Age			−0.31**	−0.33*	−0.27(*)
	Gender			−0.14	−0.12	−0.09
		Age of onset			0.10	0.11
		General functioning			0.19(*)	0.09
			Self‐concept clarity			0.26*
Model change (Step 1–Step 3)						
*F*				6.37**	4.42***	4.77***
Δ*R* ^2^				0.13**	0.04	0.05*
Adj. *R* ^2^				0.11	0.13	0.17

(*) *p* = 0.06.

* *p* < 0.05.

** *p* < 0.01.

*** *p* < 0.001.

## 4 Discussion

In this study, a large sample of adults living with SZ completed a mixed‐measurement design that examined how different aspects of temporal sense of self—self‐concept clarity and future narrative identity—relate to future outlook (i.e., hope and perceived influence of diagnosis). The results showed that both aspects of the temporal self contributed uniquely albeit in different ways: self‐concept clarity independently predicted both greater hope and reduced perceived diagnostic influence, whereas future narrative identity characterized by a positive tone and themes of communion uniquely predicted hope. We will elaborate on these findings in what follows.

### 4.1 The Temporal Self in SZ and Outlook on the Future

The present findings can be understood within the broader framework of schizophrenia as a disorder of the self (Lysaker and Lysaker 2010; Sass and Parnas 2003). Difficulties with self‐concept clarity and narrative identity reflect theoretically distinct disruptions in the temporal self (see also Dunlop 2018; McAdams 2018). The former refers to how clearly defined, coherent, and stable the self is over time, while the latter reflects the ability to integrate past, present, and future into a meaningful whole. This study focused on how individuals project their present self into the future—a dimension that has received little empirical attention. Our results suggest that although the two constructs are conceptually related, they influence how individuals with schizophrenia perceive their future in different ways: they were not significantly correlated, and each contributed uniquely to the prediction of hope and the perceived centrality of diagnosis.

The finding that self‐concept clarity was associated with both greater hope for the future and a reduced perceived influence of the diagnosis suggests that a clear and coherent sense of past and present self enables individuals to envision a future not entirely defined by illness. From a recovery perspective, this underscores the potential value of interventions that aimed at strengthening self‐concept clarity to promote optimism and agency (Thomsen et al. 2023).

Moreover, future narrative identity, specifically the emotional tone and themes of communion, was uniquely related to hope. The link between communion and a hopeful outlook aligns with research emphasizing the importance of social connection, belonging, and supportive relationships in the recovery process (Bjørlykhaug et al. 2022; Wang et al. 2018). By contrast, the correlation analysis showed that none of the aspects of future narratives were related to perceived diagnostic influence. This may appear somewhat surprising, as agency captures themes of autonomy and control, and might therefore be expected to relate to a future perspective less defined by illness. It may be explained by prior research showing that, compared to past narratives, future narratives are often more idealized, optimistic and “scripted” (e.g., Jensen et al. 2021; Thomsen et al. 2023). Such narratives likely foster hope but may be less relevant when evaluating the long‐term impact of the diagnosis, possibly explaining the nonsignificant relations between future narrative identity and perceived influence of the diagnosis. It is also possible that some individuals, while imagining agentic futures, continue to acknowledge the persistent presence of their diagnosis. In contrast, self‐concept clarity, focused on the coherence and stability of the self, may provide a more solid foundation for viewing the future as less determined by illness. Of note, associations between self‐concept clarity, narrative identity, and future outlook were not explained by functional impairment or an earlier age of onset, both of which are typically associated with more pessimistic outlook.

### 4.2 Clinical Implications

The observed associations between sense of self and future outlook hold promise for scaffolding recovery in SZ. Interventions that strengthen self‐concept and identity across time and context may foster the robustness and autonomy needed to envision a hopeful future in which the diagnosis is less central. While treatment approaches focusing on strengthening the sense of self have repeatedly been encouraged to facilitate better recovery (see e.g., Lysaker et al. 2010; Møller 2023) such treatment options are typically not available to individuals with SZ for various reasons. However, interventions focusing on narrative repair—aimed at developing a coherent illness narrative while integrating alternative stories of strengths, hopes, and aspirations, and establishing stepping stones toward a hopeful yet realistic future—have been argued to be particularly meaningful for these individuals (Thomsen et al. 2023; see also mentalization‐based approaches, e.g., Debbané et al. 2016; Sanz et al. 2024).

Our results further indicate that individuals with lower functioning and, in particular, younger individuals may struggle the most with sustaining an optimistic future outlook. These findings are consistent with research linking impaired functioning with a more pessimistic future outlook (see e.g., Laukkanen et al. 2001) and suggesting that younger individuals may not yet have the life experience nor the self‐knowledge to know how to cope with future challenges adaptively (Lind et al. 2019, 2021). For these reasons, treatment focusing on strengthening different aspects of sense of temporal self seems particularly relevant for this group of individuals.

### 4.3 Limitations and Future Directions

Some study limitations should be acknowledged serving as steppingstones for optimizing future studies on this topic. First, the study is cross‐sectional, and no cause‐effect mechanisms can be determined based on study results. Thus, it is likely that a more hopeful and optimistic future outlook also influences the participants' temporal sense of self. Indeed, research suggests that past and future are interrelated constructs (e.g., Szpunar 2010) and longitudinal studies are warranted to better disentangle these relations. Second, different methods were used across measures: narrative identity and perceived influence of diagnosis were content coded, whereas self‐concept clarity and hope were self‐reported. The mixed‐measurement approach adhere to how self‐concept clarity and narrative identity are typically assessed and provide both qualitative insights and standardized self‐assessments, offering a broader perspective on participants' experiences. Yet, it also limits direct comparability across measures. In addition, the perceived influence of diagnosis was assessed with a single open‐ended question, which may lack the robustness of standardized instruments. This methodological heterogeneity may partly explain why some constructs did not correlate, as it could weaken potential associations. Moreover, only future narrative identity was measured, although previous research indicates that past life story chapters differ more between individuals with SZ and controls than future chapters do (Jensen et al. 2021). Future studies may therefore benefit from including additional aspects of narrative identity, such as the past life story (McAdams 2018) to provide a more comprehensive picture of a person's narrative identity.

Finally, while this study focused on narrative identity and self‐concept clarity in relation to future hope, it should be noted that hope is a multifaceted construct likely influenced by many additional factors (e.g., social support, mental, additional aspects of personality). The absence of data on participants' mood or affective symptoms at the time of participation is a limitation, as these can influence the participants responses and are therefore relevant to describe and account for. Further, the study did not register other psychiatric comorbidities (e.g., anxiety disorders) or substance use, which could also affect future narratives. Future research may therefore consider taking more aspects into account including comorbidity but also sociodemographic variables (e.g., income, relationship statues) to obtain a more comprehensive understanding of what impact hope in future outlook in SZ. Finally, while the sample was recruited systematically through the nationwide mental‐health organization EN AF OS (2022), future studies may replicate the findings in a sample where the SZ diagnosis is confirmed with clinical assessment. However, with targeted recruitment and the detailed replies of the participants, we have no specific reasons to question the validity of their diagnosis.

## 5 Conclusion

SZ has been referred to as a self‐disorder and the present study nuances this picture by showing how two fundamental aspects of the temporal sense of self in the form of self‐concept clarity and narrative identity both influence future outlook in SZ over and above illness‐related variables. These findings suggest that temporal self‐processes are not only theoretically relevant for understanding self‐disturbance in SZ, but also potentially clinically important for supporting recovery. Specifically, strengthening clarity and coherence of the self, as well as supporting more positive and socially embedded future narratives, may represent potential targets for interventions that foster hope and counter the hopelessness linked to poorer outcomes in SZ.

## Author Contributions

**Majse Lind:** conceptualization, methodology, formal analysis, writing – original draft. **Ragnhild Svendsen:** data curation, investigation, writing – review and editing. **Kristina Flesjø:** data curation, investigation, writing – review and editing. **Kristine Kahr Nilsson:** conceptualization, methodology, formal analysis, writing – review and editing.

## Funding

The authors have nothing to report.

## Ethics Statement

The North Denmark Region Committee on Health Research Ethics. Qualifies survey‐based studies as exempt for further approvals. Informed consent was obtained from all the participants, and data were treated in accordance with the Danish Data Protection Act.

## Consent

Informed consent was obtained from all the participants.

## Conflicts of Interest

The authors declare no conflicts of interest.

## Data Availability

Data can be made available upon request to the first or senior author.
